# Stress-Dose Steroids: A Potential Therapeutic Option for Refractory Hyperkalemia

**DOI:** 10.7759/cureus.44770

**Published:** 2023-09-06

**Authors:** Pooya Zardoost, Zeryab Khan, Rachel Kim, Kelsey Scott, Henry L Wehrum

**Affiliations:** 1 Internal Medicine, OhioHealth Doctors Hospital, Columbus, USA; 2 Graduate Medical Education, OhioHealth Doctors Hospital, Columbus, USA

**Keywords:** general nephrology dialysis and transplantation, sepsis, acute kidney injury, general nephrology, nephrology, potassium, systemic steroids, steroids, stress dose of steroids, refractory hyperkalemia

## Abstract

Hyperkalemia refractory to standard temporization measures can be life-threatening, and urgent hemodialysis is often utilized as a final resort. Our patient presented with hyperkalemia that was multifactorial in etiology, with acute kidney injury complicated by adrenal insufficiency. Her hyperkalemia was refractory to temporization and excretion agents, and hemodialysis was being considered. Given a recent infection, surgery, and borderline hypotension with low adrenocorticotropic hormone, there was a concern for adrenal insufficiency. However, a full investigation for secondary adrenal insufficiency via magnetic resonance imaging could not be conducted as the patient suffered from claustrophobia. Continued concern for adrenal insufficiency prompted the initiation of intravenous hydrocortisone, and the patient’s hyperkalemia resolved within 24 hours. While suspected adrenal insufficiency is already a basis for stress-dose steroids in the setting of pathologies such as severe sepsis, clinicians should have a low threshold for considering refractory hyperkalemia alone as an indication for stress-dose steroids. When dialysis is being considered as an option, this treatment modality should be given even more consideration. Adopting this practice may not only lead to improved mortality from hyperkalemia but also lead to fewer patients being exposed to the risks of dialysis.

## Introduction

Hyperkalemia can be life-threatening as displacement of this predominantly intracellular cation can lead to fatal cardiac arrhythmia [1-3]. Successful management of this metabolic derangement requires a careful approach that consists of treatments that shift potassium intracellularly, stabilize the membrane of cardiac myocytes, and promote excretion. Reduced mineralocorticoid levels cause impaired potassium secretion [1]. We had a patient in our facility whose potassium levels remained high despite attempts to promote intracellular shift and excretion, and dialysis was being considered as a final option. Stress-dose steroids led to the resolution of hyperkalemia. The potential utility of stress-dose steroids for refractory hyperkalemia deserves further attention.

This article was previously presented as a poster abstract at the Society of Hospital Medicine Converge on March 29, 2023.

## Case presentation

**Figure 1:**
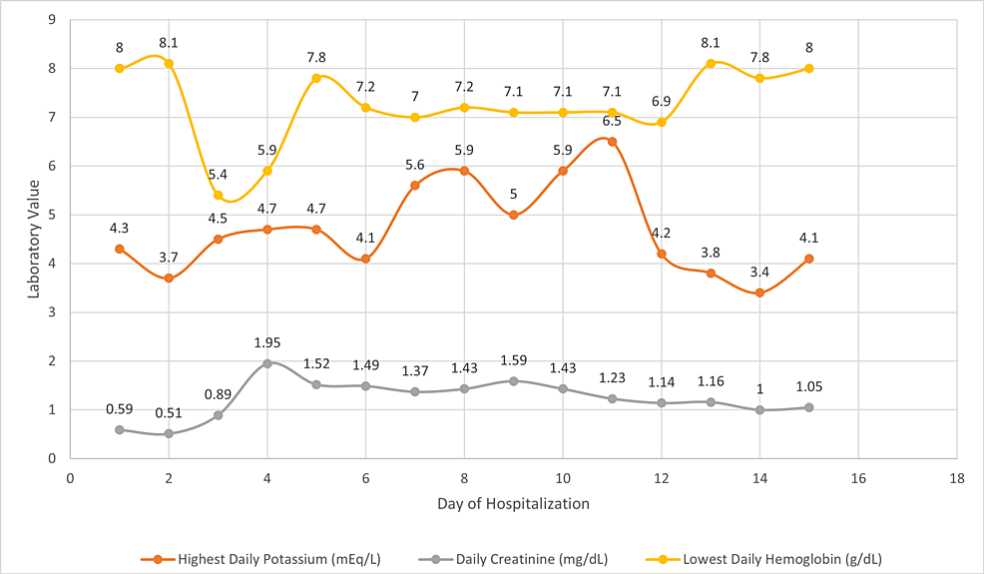
Patient’s peak potassium, creatinine, and lowest hemoglobin based on the day of hospitalization. Her debridement procedure was on day two. Her hemoglobin dropped from 8.1 g/dL to 6.3 g/dL which recovered to baseline after four units of packed red blood cell transfusions, two of which were given on day 3 and the other two on day 4. Temporizing agents included albuterol nebulizer solution at 10 mg, insulin regular at 10 U with 10% dextrose in 250 mL free water, and excretion agents consisting of furosemide 40 mg and sodium-zirconium-cyclosilicate at 10 g oral. She also received 1 g of calcium gluconate when her potassium peaked above 6 mEq/L. Note the rise in serum creatinine on day four after the acute blood loss anemia as well as the persistence of hyperkalemia after improvement in serum creatinine. Day 11 is when stress-dose steroids were initiated.

A 48-year-old female with a history of opioid use disorder, hypertension, and recent right hip arthroplasty presented with complaints of fevers and purulent drainage at her surgical site. Home medications included lisinopril 20 mg daily and propranolol 80 mg daily. Presenting vital signs consisted of a blood pressure of 137/77 mmHg, a heart rate of 80 beats per minute, a respiratory rate of 18 breaths per minute, a temperature of 99.8°F, and oxygen saturation of 96% on room air. Pertinent examination findings included tenderness and erythema on her right hip. Her lungs were clear to auscultation bilaterally with no wheezes, rales, or rhonchi. Her cardiovascular examination revealed a regular rate and rhythm, with a capillary refill of fewer than two seconds. Initial labs including white blood cell count, platelets, and lactic acid were within normal limits, as were creatinine and potassium (Figure 1). Her hemoglobin was 8.1 mg/dL and the workup revealed iron deficiency anemia, for which supplementation was initiated. Her home antihypertensives were not continued due to normotensive blood pressures and concern for impending volume shift given planned procedural interventions. Intravenous (IV) vancomycin was initiated, and she underwent irrigation and debridement of her right hip with cultures collected. The day following the procedure, labs revealed worsened anemia, acute kidney injury (AKI), and mild hypotension with systolic blood pressure in the 90-100 mmHg range. Mean arterial pressure remained above 65 mmHg. There were no temporal variations throughout her admission. Her examinations both before and after the procedure were not concerning for her anemia, as there was no tachycardia or pallor. Strict intake and output measurements were initiated, and the patient was consistently non-oliguric with output ranging from 1 to 2 L daily at 0.5 to 1 cc per kg/hour.

Treatment included lactated Ringer’s (LR) infusion at 100 mL/hour as well as boluses and blood transfusions with a total of four units of packed red blood cells. The patient’s anemia, blood pressure, and creatinine began to improve. However, the patient began to develop hyperkalemia which persisted despite improvement in renal function as well as multiple treatments with IV insulin, sodium polystyrene sulfonate, albuterol nebulizer and furosemide. Creatinine phosphokinase (CPK) was within normal range, and there were no difficulties with phlebotomy` (Table 1). Workup included transtubular potassium gradient of 2 which, in the setting of hyperkalemia, raised concern for hypoaldosteronism and adrenal insufficiency. Aldosterone level was decreased, and renin was within normal limits, as shown in Table 1. Additional lab work revealed low serum adrenocorticotropic hormone (ACTH) and random cortisol within normal limits. Nephrology and endocrinology were consulted for assistance. Further history revealed the patient had a diet abundant in potatoes and orange juice, which she was consuming during hospitalization. Her potassium remained high despite being transitioned to a potassium restricted diet.

**Table 1:** Investigative lab values. *: Adrenocorticotropic hormone values in our facility below 1.5 pg/mL are listed as <1.5. **: Aldosterone values in our facility less than 4 ng/dL are listed as <4.0. ***: The initial cortisol level before cosyntropin was administered was not collected; however, as the 30-minute value was above 18 µg/dL, this was interpreted as a sufficient adrenal response.

Laboratory value	Reference range	Patient’s value
Adrenocorticotropic hormone (pg/mL)	7.2–63.3	<1.5*
Random cortisol (µg/dL)	5–25	7.5
Renin (ng/mL/hour)	0.6–3	1.2
Aldosterone (ng/dL)	3.1–35.4	<4.0**
Cosyntropin test (µg/dL)	N/A	30 minutes: 18.4, 60 minutes: 24.1***
Creatinine phosphokinase (U/L)	40–170 U/L	46

**Figure 2:**
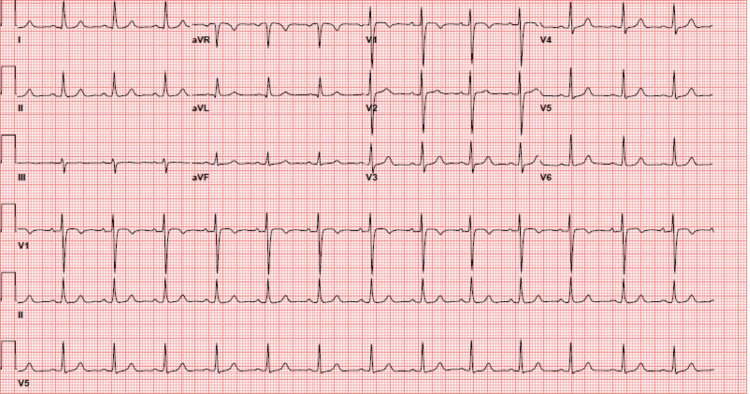
Electrocardiogram of the patient during the rise in potassium. Normal sinus rhythm electrocardiogram of the patient on day 9 of hospitalization. Two other electrocardiograms during admission are similar in interpretation. Note the lack of peaked T waves, QRS widening, or flattened PR interval.

Cosyntropin stimulation test revealed an intact adrenal response. Recent infection in the setting of acute blood loss anemia, hypotension, and hyperkalemia prompted concern for secondary adrenal insufficiency, and an MRI was ordered to rule out a pituitary adenoma, but the patient suffered from claustrophobia and the imaging study was not successfully done. Nephrology initiated a sodium bicarbonate infusion and was also considering hemodialysis if the potassium did not improve the next day, as by now it had reached a peak of 6.5 mEq/L (Figure 1). Electrocardiograms persistently revealed normal sinus rhythm and no segmental or wave abnormalities (Figure 2). Intravenous hydrocortisone at 100 mg every eight hours was started per endocrinology, and by the next day, the hyperkalemia resolved, and her creatinine returned to baseline in the next 48 hours. The sodium bicarbonate infusion was discontinued, and the hydrocortisone was tapered down gradually to intravenous 50 mg twice a day, followed by 25 mg twice a day, and then an oral regimen for a total of four days. Wound cultures from the hip irrigation returned positive for methicillin-resistant Staphylococcus aureus (MRSA), and the patient was discharged and completed a one-month course of IV daptomycin at a short-stay unit. The patient received follow-up labs one month later, including adrenal labs that included ACTH and random cortisol that were within normal limits.

## Discussion

The cause of our patient’s hyperkalemia was rooted in a combination of several factors. Hyperkalemia has many potential causes, including cellular redistribution from acidosis or injury such as in rhabdomyolysis, excess intake in the setting of impaired renal potassium handling, decreased renal excretion via decreased distal delivery (such as in oliguric renal failure), mineralocorticoid deficiency, and tubular defects [2]. The etiology of our patient’s hyperkalemia was a combination of AKI and adrenal insufficiency, with both stemming from volume loss, surgery, and infection.

While our patient did have a recent hip arthroplasty, she did not present initially with hyperkalemia, which made trauma from the previous surgery an unlikely cause. A normal CPK level ruled out rhabdomyolysis. Although the patient’s home medications included lisinopril, this was not continued, and she was not placed on any medications during admission that affect potassium homeostasis. Antihypertensives such as an angiotensin-converting enzyme inhibitor, angiotensin II receptor blocker, or direct renin inhibitor such as aliskiren can contribute to hyperkalemia [1]. Nonsteroidal anti-inflammatory drugs reduce distal delivery of sodium to the nephron and suppress renin release as a result, which was not administered for analgesia [1]. Potassium-sparing diuretics such as spironolactone and amiloride also can cause hyperkalemia, either by competing with aldosterone for the epithelial sodium channel in the principal cell of the distal nephron or by inhibiting it [1].

While pseudo-hyperkalemia is also a cause of elevated potassium values, there were no reported issues from phlebotomy and the patient did not have an elevated white blood cell or platelet count. The patient reported consuming food products high in potassium such as potatoes and orange juice, and this was suspected to have played an exacerbating role as excess intake usually becomes an issue when there is a defect with renal excretion of potassium [2]. The patient’s low transtubular potassium gradient of 2 in the setting of hyperkalemia, lower range of normal renin level, low aldosterone level, and normal cortisol studies were consistent with hyporeninemic hypoaldosteronism. In the absence of hyperglycemia, this also raised concern for adrenal insufficiency [4].

The patient’s renal function, while improving after fluids, was still not resolved to the patient’s baseline creatinine of 0.5-0.6 mg/dL, which was a driving force behind the patient’s hyperkalemia. Reduced kidney function causes hyperkalemia in proportion to a decrease in glomerular filtration rate [2,5]. Urinary creatinine or electrolyte studies were not collected to calculate the fractional excretion of sodium, but the patient’s recent surgical history including improvement in creatinine after fluids raised suspicion for a pre-renal etiology to the AKI.

While literature remains conflicted on mortality benefits, glucocorticoids are commonly used in patients with severe sepsis [6,7]. It is postulated that cortisol production is not able to meet the higher metabolic demands of sepsis, resulting in relative adrenal insufficiency; the phenomenon is also termed critical illness-related corticosteroid insufficiency [8]. While our patient did not meet the criteria for systemic inflammatory response syndrome despite confirmed infection, hypotensive vitals during admission as well as physiological stressors of infection and blood loss anemia status post-surgery raised suspicion for adrenal insufficiency playing a role in the patient’s hyperkalemia. The patient’s hypotension was directly associated with blood loss anemia; this contributed to a relative adrenal insufficiency and pre-renal AKI. The cosyntropin test revealed intact adrenal function, but secondary or relative adrenal insufficiency was not completely ruled out. In addition to investigating excess metabolic demands in the setting of infection and trauma, one must rule out an underlying mass effect influencing secretion in the hypothalamus-pituitary axis [9]. Barriers to investigation via MRI hindered a comprehensive investigation of secondary adrenal insufficiency, but continued concern in the setting of hyperkalemia and hypotension warranted immediate intervention. The level of hyperkalemia specifically, despite attempts to temporize and increase potassium excretion, also underscored the urgency.

Studies have shown that hydrocortisone has a 1:1 equivalent mineralocorticoid activity, and administration of this regimen led to the resolution of the patient’s hyperkalemia and prevented the patient from receiving dialysis [10]. Activation of mineralocorticoid receptors via aldosterone leads to increased activity of the sodium/potassium-adenosine phosphatase pump and epithelial sodium channel in the distal tubules of the kidney, leading to an electrochemical gradient that drives potassium into the lumen, and the resulting effect is increased sodium reabsorption and potassium secretion [2,5]. Cortisol also affects potassium via a mineralocorticoid-like effect, which is underscored by the 1:1 equivalent activity [11].

A potential limiting factor in our observation is the administration of sodium bicarbonate on the same day as hydrocortisone administration. However, sodium bicarbonate was chosen as a treatment option after several other treatment methods failed to lower serum potassium. Our patient did not have metabolic acidosis, and most of the literature showing a hypokalemic effect of sodium bicarbonate involves metabolic acidosis [2]. Nevertheless, small studies have shown a hypokalemic effect of sodium bicarbonate irrespective of pH, and the multidisciplinary team’s focus with our patient was to prevent the risks of hemodialysis, which warranted a trial of this treatment modality [2].

Additional factors that need to be addressed are the maintenance fluid of choice as well as blood product administration [3]. Regarding the former contributing factor, LR contains 4 mEq/L of potassium, and providers often refrain from using this crystalloid solution when there is a risk of hyperkalemia such as reduced kidney function [12]. However, current evidence on the clinical impact of this phenomenon is a topic of debate among clinicians including nephrologists [12]. A 2022 retrospective cohort-based observational study included 293 clinical encounters who had an estimated glomerular filtration rate of less than 30 mL/minute and had received a minimum of 500 mL of LR during the admission [12]. LR use was not independently associated with the development of hyperkalemia in patients with reduced kidney function [12]. While hemolysis and cellular shift from multiple transfusions are often a concern among clinicians, red blood cell transfusions contribute to serum potassium, even after the use of LR was non-significant [3,12]. While these treatments may have complicated the course of the patient’s serum potassium, there were other factors at play, such as the patient’s relative adrenal insufficiency, acute blood loss anemia, and AKI that required management.

## Conclusions

Hyperkalemia can precipitate fatal arrhythmias, and identifying the etiology and initiating management is crucial. Literature suggests that cortisol acts on potassium via a mineralocorticoid-like effect, and adrenal insufficiency is an important and often overlooked etiology of hyperkalemia. The patient was empirically treated with a course of high-dose IV steroids with excellent response and subsequent normalization of hyperkalemia. While adrenal insufficiency is an indication for steroid therapy in current clinical practice, the metabolic derangement of hyperkalemia alone should be considered as a basis for this therapy, especially if it can result in avoiding dialysis.
